# Comparative evaluation of left ventricular mass regression after aortic valve replacement: a prospective randomized analysis

**DOI:** 10.1186/1749-8090-6-136

**Published:** 2011-10-13

**Authors:** Mirko Doss, Jeffrey P Wood, Arndt H Kiessling, Anton Moritz

**Affiliations:** 1 Department of Thoracic and Cardiovascular Surgery, Johann Wolfgang Goethe University, Frankfurt am Main, Germany

**Keywords:** Left Ventricular Mass, Aortic Valve Replacement, Prospective randomized Analysis

## Abstract

**Background:**

We assessed the hemodynamic performance of various prostheses and the clinical outcomes after aortic valve replacement, in different age groups.

**Methods:**

One-hundred-and-twenty patients with isolated aortic valve stenosis were included in this prospective randomized randomised trial and allocated in three age-groups to receive either pulmonary autograft (PA, n = 20) or mechanical prosthesis (MP, Edwards Mira n = 20) in group 1 (age < 55 years), either stentless bioprosthesis (CE Prima Plus n = 20) or MP (Edwards Mira n = 20) in group 2 (age 55-75 years) and either stentless (CE Prima Plus n = 20) or stented bioprosthesis (CE Perimount n = 20) in group 3 (age > 75). Clinical outcomes and hemodynamic performance were evaluated at discharge, six months and one year.

**Results:**

In group 1, patients with PA had significantly lower mean gradients than the MP (2.6 vs. 10.9 mmHg, p = 0.0005) with comparable left ventricular mass regression (LVMR). Morbidity included 1 stroke in the PA population and 1 gastrointestinal bleeding in the MP subgroup. In group 2, mean gradients did not differ significantly between both populations (7.0 vs. 8.9 mmHg, p = 0.81). The rate of LVMR and EF were comparable at 12 months; each group with one mortality. Morbidity included 1 stroke and 1 gastrointestinal bleeding in the stentless and 3 bleeding complications in the MP group. In group 3, mean gradients did not differ significantly (7.8 vs 6.5 mmHg, p = 0.06). Postoperative EF and LVMR were comparable. There were 3 deaths in the stented group and no mortality in the stentless group. Morbidity included 1 endocarditis and 1 stroke in the stentless compared to 1 endocarditis, 1 stroke and one pulmonary embolism in the stented group.

**Conclusions:**

Clinical outcomes justify valve replacement with either valve substitute in the respective age groups. The PA hemodynamically outperformed the MPs. Stentless valves however, did not demonstrate significantly superior hemodynamics or outcomes in comparison to stented bioprosthesis or MPs.

## Background

Aortic stenosis is the predominant lesion in the majority of patients presenting with clinically significant aortic valve disease. The only definitive treatment of critical aortic stenosis is aortic valve replacement (AVR).

In deciding the choice of prosthesis in simple aortic valve replacement, most surgeons recommend a mechanical valve in the younger patients and a stented bioprosthesis in older individuals. Within the last decade, pulmonary autografts and stentless bioprosthesis have been established as alternatives to mechanical valves and stented bioprosthesis, respectively. The precise age at which one prosthesis is preferred over the other is a matter of controversy, but recent studies indicate that patients over the age of 65 years should receive a bioprosthesis (stented or stentless) and patients whose life expectancy is at least 15 years should receive a mechanical valve [1]. The latter group of patients would alternatively be eligible for a pulmonary autograft. Stentless valves with their unique design features, that allow laminar flow resulting in less stress on leaflets, promise an even longer freedom from structural valve deterioration than stented bioprosthesis.

We can therefore identify a third group of patients, between the ages of 55-75 years that would be eligible for either a stentless bioprosthesis or a mechanical valve. Any evaluation of optimal prostheses cannot be based on durability data alone, and must include hemodynamic assessment and clinical performance of the valvular substitutes, judged according to the "guidelines for reporting morbidity and mortality after cardiac valvular operations."[2].

Regression of LV-hypertrophy after AVR, being one of the key determinants of postoperative morbidity and mortality, has been under investigation by many groups in the field. The literature provides extensive documentation on non randomized assessment of mechanical and bioprosthesis, with regards to LV mass regression. However, few prospective randomized clinical studies are reported.

The aim of the current study was to provide some rationale to select the optimal valve substitute, for a certain age group, based on valve performance and its effects on regression of LV hypertrophy in a prospective randomized setting.

## Methods

One-hundred and twenty patients undergoing elective aortic valve replacement were entered in this prospective evaluation. Dependent on their age, they were allocated into tree groups. Patients in group I were less than 55 years of age and were randomized to receive either a mechanical (Edwards Mira, n = 20) or a pulmonary autograft (n = 20). Patients in group II were between 55-75 years old and were randomized for a stentless bioprosthesis (CE Prima Plus, n = 20) or a mechanical valve (Edwards Mira, n = 20). Patients in group III were above the age of 75 years and were randomly assigned to a stentless bioprosthesis (CE Prima Plus, n = 20) or a stented bioprosthesis (CE Perimount, n = 20). All patients underwent preoperative and postoperative transthoracic echocardiography (at discharge, 6 and 12 months) for functional and structural assessment. All clinical and echocardiographic data describing this population were prespecified and collected postoperatively. A valvular database, provided by Edwards Lifesciences, was used to collect preoperative, perioperative and postoperative patient information. The study protocol was approved by our institutional ethics review board. All patients provided written informed consent before entering the study.

The choice of valve prosthesis was made preoperatively and feasibility of implantation was confirmed intraoperatively. Severe calcification of the aortic root diagnosed intraoperatively, very low position of coronary ostia in relation to the annulus and atypical insertion of the coronary ostia made it impossible to implant stentless valves or pulmonary autografts.

**Figure 1:**
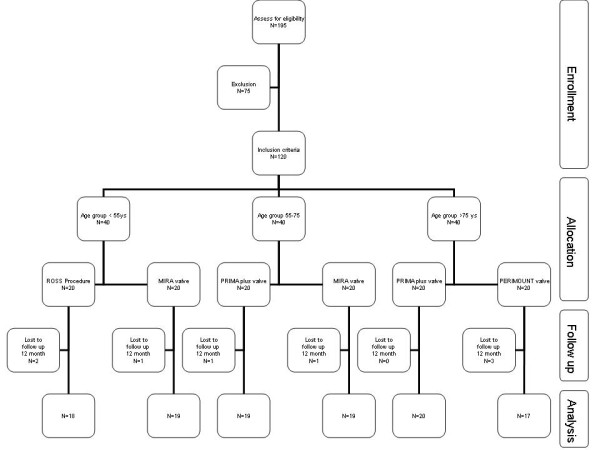
**Recruitment flow chart**.

Preoperative transthoracic echocardiography was used to identify patients in whom the use of stentless valves and pulmonary autografts seemed safe. The sizes of both the native aorta and at the level of the sinotubular junction and the annulus were measured. If the diameter of the annulus was found to be larger than or less than the size of the sinotubular junction by more than 2 valve sizes (i.e. 4 mm), patients were excluded from the study. Patients with a subvalvular pressure gradient, active endocarditis and the need for concomitant valvular surgery were similarly excluded from enrolment.(Figure 1)

### Operative technique

Access to the heart was gained via median sternotomy. Standard extracorporeal circulation with moderate hypothermia (28°C) was used. All patients had antegrade and retrograde cold blood cardioplegia and carbon dioxide insufflation of the open thorax for organ protection.

All pulmonary autograft procedures were performed as root replacements with implantation of the coronary arteries on the graft. Reconstruction of the right ventricular outflow tract was performed with cryopreserved pulmonary valve homografts in all patients.

The Prima Plus stentless bioprosthesis were implanted in the subcoronary position. The aortic valve was exposed via a transverse aortotomy. After resection of the native aortic valve and debridement of the aortic annulus, accurate sizing was carried out using the respective seizers. The commissures were positioned 120° apart with the muscular shelf corresponding to the right coronary sinus. Single interrupted unpledgeted 4-0 Ethibond sutures were used for the proximal end and the rims of the valve commissures were sutured to the native aorta using 4-0 polypropylene running sutures.

For the Mira mechanical aortic valves and the Perimount stented bioprosthesis access to the aortic valve was gained via a hockeystick aortotomy. The valves were implanted in the supraannular position. Interrupted mattressed pledgeted 2-0 Ethibond sutures were placed circumferentially from below the annulus. Mechanical valves were oriented in the antianatomical position.

### Echocardiography

Two experienced operators performed all echocardiograms for the study on a standard machine (System Five, Sonotron Vingmed). Cardiac morphology and function as well as hemodynamic parameters were assessed. All hemodynamic measurements were performed with patients in stable conditions. Aortic valve flow velocities were assessed with continuous wave Doppler. End diastolic left ventricular posterior wall thickness > 12 mm was considered hypertrophied. Aortic valve incompetence was judged as transvalvular or paravalvular and graded according to the regurgitant jet area in relation to left ventricle as mild, moderate or severe. Apart from standard imaging views, preoperative echocardiography also included the measurement of the diameter of the native aortic annulus and the sinotubular junction as well as the assessment of subvalvular gradients, in order to identify a possible mismatch between annulus and sinotubular junction or excessive subvalvular hypertrophy. Both conditions would render the patient unsuitable for the study.

### Follow up

Follow up examinations were scheduled for discharge from the hospital, at six and 12 months postoperatively. All patients were subject to detailed clinical and echocardiographic follow-up. This included the New York Heart Association functional class (NYHA), blood data including signs of haemolysis, anticoagulation profile, assessment of cardiac rhythm and blood pressure and documentation of occurrence of early and late complications.

In echocardiography follow-up, our special attention was focused on the regression of LV-hypertrophy. Both completeness and rate of LV-mass regression ware assessed. In addition, changes in LV-function and hemodynamics including effective orifice area (EOA), as well as changes in postoperative transvalvular gradients were analyzed.

### Anticoagulation regime

Our anticoagulation regime was as follows. Patients with pulmonary autografts did not receive oral anticoagulation. Patients with bioprosthesis had oral anticoagulation for 3 months and patients with mechanical valves had lifelong anticoagulation.

Our protocol included subcutaneous low molecular heparin for the first day and parallel oral anticoagulation with vitamin K antagonists. As soon as the International Normalized Ration (INR) levels reached the therapeutic target range of 2.5 - 3.5, the heparin was discontinued. Initially, oral anticoagulation was monitored by the patient's general practitioners. However, most patients who received mechanical valves soon attended a structured course on oral anticoagulation self management, and henceforth monitored their own INR levels, using the portable CoaguCheck™ (Roche Diagnostics) device.

### Statistical methods

All data were compiled and analyzed using Microsoft Access, Microsoft Excel (Redmont WA) and StatView (Cary, NC). The baseline characteristics and hospital outcomes for the two groups were compared using chi-square or Fisher's exact test for categorical data and unpaired t-tests for continuous variables. Results are reported as mean ± standard deviation in text and tables. Statistical significance was defined as a p value less than 0.05.

## Results

Patients were allocated according to their age and therefore results are reported separately for the respective age groups.

### Group I (*pulmonary autograft vs. mechanical valve replacement, age < 55 years*)

The two patient groups were comparable with regards to preoperative demographic data and clinical characteristics (table 1). Cross-clamp times and total cardiopulmonary bypass times were significantly longer in the pulmonary autograft group. A summary of intraoperative outcomes is given in Table 2. There were no intraoperative deaths and all patients were transferred to the intensive care unit in stable conditions. Rethoracotomy for bleeding had to be performed in 3 patients, all in the mechanical group. None of these patients required prolonged mechanical ventilation and had an uneventful recovery.

**Table 1:** Preoperative patient characteristics

	ROSS n = 20	MIRA n = 20	PRIMA PLUS n = 20	MIRA n = 20	PRIMA PLUS n = 20	PERI- MOUNT n = 20
Female	8	9	11	10	9	12

Male	12	11	9	10	11	8

Age (years)	49 ± 8.3	48 ± 6.9	60 ± 4.1	62 ± 2.3	78 ± 3.8	79 ± 4.3

BSA (m^2^)	1.82 ± 0.7	1.89 ± 0.4	1.76 ± 0.2	1.67 ± 0.2	1.79 ± 0.6	1.85 ± 0.8

Hypertension	7	6	9	8	10	9

Atrial fibrillation	0	1	0	0	0	1

Mean gradient (mmHg)	56.6 ± 12.6	59.4 ± 16.2	49.0 ± 20.0	52.0 ± 18.0	58.1 ± 18.2	50.9 ± 14.8

IVS (cm)	1.98 ± 0.2	1.82 ± 0.4	1.95 ± 0.3	1.97 ± 0.2	1.94 ± 1.9	1.91 ± 0.9

LVW (cm)	1.95 ± 0.3	1.81 ± 0.2	1.99 ± 0.5	2.01 ± 0.4	1.93 ± 0.3	1.98 ± 0.2

LVEDD (cm)	4.9 ± 0.5	4.7 ± 0.3	4.6 ± 0.3	4.8 ± 0.3	4.8 ± 0.4	4.6 ± 0.3

LVESD (cm)	3.7 ± 0.4	3.4 ± 0.3	3.6 ± 0.2	3.9 ± 0.4	3.2 ± 0.3	3.5 ± 0.2

PEF EF (%)	66.3 ± 7.9	67.2 ± 6.8	62.0 ± 7.0	65.0 ± 6.0	65.9 ± 7.4	66.6 ± 8.6

NYHA III-IV	16	16	17	15	17	18

Concomitant CABG	0	2	0	0	2	3

**Table 2:** Intraoperative outcomes

	ROSS n = 20	MIRA n = 20	PRIMA PLUS n = 20	MIRA n = 20	PRIMA PLUS n = 20	PERI- MOUNT n = 20
Cross-clamp time (min)	111 ± 21	75 ± 19	102 ± 22	76 ± 24	108 ± 17	79 ± 17

CPB time (min)	141 ± 37	102 ± 23	128 ± 26	104 ± 19	130 ± 19	105 ± 23

Implantation technique	full root	supraannular	subcoronary	supraannular	subcoronary	supraannular

Subaortic stenosis	0	0	0	0	0	0

Mean annular diameter (mm)	25 ± 3.6	22 ± 1.6	22.4 ± 1.9	22.9 ± 1.8	21.2 ± 1.6	20.6 ± 1.9

Mean valve size implanted (mm)	24.9 ± 2.3	24.2 ± 1.7	24.1 ± 1.8	24.8 ± 1.5	23.9 ± 1.8	22.8 ± 1.9

						

Valve Size (mm)						

21	0	0	5	0	4	6

23	6	11	7	10	9	10

25	8	9	5	7	6	4

27	3	0	3	3	1	0

29	2	0	0	0	0	0

There were no perioperative deaths in either group and all patients were discharged from hospital. At follow-up, two late deaths had occurred in the pulmonary autograft group. Both patients died at home and sudden death was suspected by the general practitioner, although the cause of death was not confirmed at autopsy. There was one late death in the mechanical valve group. After being admitted to hospital due to pneumonia this patient required intubation and mechanical ventilation. Eventually the patient died of sepsis.

Another two patients in the pulmonary autograft group required reoperation for aortic root dilatation and subsequent severe aortic regurgitation. Both patients received mechanical heart valves 7 and 11 months after their initial procedure.

There was one anticoagulation-related complication in the mechanical valve group. The patient had a gastrointestinal bleeding and required hospitalisation. One patient in the pulmonary autograft group suffered a stroke 6 months after surgery. At the time he was in sinus rhythm and underwent an intensive search for what might have caused this stroke. However, other than his recent aortic valve surgery, no other risk factors could be identified. There were no other valve related complications in this group. Hemodynamic performance was significantly better in the pulmonary autograft group. The LV mass regression however did not differ significantly between the groups. All echocardiographic data regarding regression of LV mass, ejection fraction, transvalvular gradients and effective orifice area are summarized in table 3.

**Table 3:** Echocardiographic findings

	ROSS n = 20	MIRA n = 20	PRIMA PLUS n = 20	MIRA n = 20	PRIMA PLUS n = 20	PERI-MOUNT n = 20
Mean gradient (mmHg)						

Preoperative	56.6 ± 12.6	59.4 ± 16.2	49.0 ± 20	52.0 ± 18	58.1 ± 18.2	50.9 ± 14.8

6 months	3.2 ± 1.7	9.3 ± 4.5	9.3 ± 6.9	10.2 ± 5.4	8.4 ± 3.6	7.3 ± 3.7

12 months	2.6 ± 1.3	9.3 ± 3.6	7.0 ± 4.7	8.9 ± 6.1	7.4 ± 4.9	6.6 ± 2.3

p-value	p = 0.005		p = NS		p = NS	

Effective Orifice Area (cm^2^)						

preoperative	0.71 ± 0.3	0.82 ± 0.2	0.85 ± 0.4	0.79 ± 0.3	0.87 ± 0.4	0.76 ± 0.3

6 months	2.10 ± 0.5	1.61 ± 0.4	1.70 ± 0.5	1.68 ± 0.4	1.63 ± 0.4	1.51 ± 0.6

12 months	2.50 ± 0.6	1.81 ± 0.3	1.91 ± 0.7	1.84 ± 0.5	1.83 ± 0.6	1.92 ± 0.8

p-value	p = 0.005		p = NS		p = NS	

EF (%)						

preoperative	66.3 ± 7.9	67.2 ± 6.8	62.1 ± 7.2	65.0 ± 6.1	65.9 ± 7.4	66.6 ± 8.6

6 months	66.4 ± 8.3	65.8 ± 7.8	65.4 ± 6.9	64.3 ± 7.2	67.6 ± 8.7	66.2 ± 10.5

12 months	67.5 ± 8.1	65.0 ± 10.7	66.9 ± 8.1	65.9 ± 6.8	66.6 ± 8.1	64.7 ± 11.2

p-value	p = NS		p = NS		p = NS	

Left Ventricular Posterior Wall Thickness (cm)						

preoperative	1.95 ± 0.3	1.81 ± 0.2	1.99 ± 0.5	2.01 ± 0.4	1.93 ± 0.3	1.98 ± 0.2

6 months	1.53 ± 0.3	1.45 ± 0.1	1.61 ± 0.3	1.65 ± 0.2	1.63 ± 0.2	1.66 ± 0.1

12 months	1.32 ± 0.2	1.24 ± 0.2	1.28 ± 0.2	1.24 ± 0.2	1.26 ± 0.2	1.32 ± 0.2

p-value	p = NS		p = NS		p = NS	

Interventricular Septum Thickness (cm)						

preoperative	1.98 ± 0.2	1.82 ± 0.4	1.95 ± 0.3	1.97 ± 0.2	1.94 ± 1.9	1.91 ± 0.9

6 months	1.70 ± 0.2	1.60 ± 0.2	1.60 ± 0.2	1.69 ± 0.3	1.54 ± 0.2	1.51 ± 0.2

12 months	1.34 ± 0.1	1.24 ± 0.1	1.29 ± 0.3	1.32 ± 0.2	1.24 ± 0.3	1.28 ± 0.2

p-value	p = NS		p = NS		p = NS	

Left Ventricular Mass Index (g/m^2^)						

preoperative	185 ± 42.3	179 ± 38.6	181 ± 40.9	182 ± 39.2	174 ± 34.3	180 ± 40.5

6 months	149 ± 34.1	141 ± 35.4	143 ± 34.2	145 ± 32.8	130 ± 31.0	132 ± 36.1

12 months	114 ± 27.2	110 ± 30.2	109 ± 29.3	111 ± 27.6	104 ± 28.5	106 ± 32.5

p-value	p = NS		p = NS		p = NS	

### Group II (*stentless bioprosthesis vs. mechanical valve replacement, age 55-75 years*)

Again, the two patient groups were comparable in clinical characteristics and preoperative demographics data (table 1). The cross-clamp and cardiopulmonary bypass times were longer in the stentless valve group. The intraoperative outcomes are listed in table 2. There were no intraoperative deaths. Early postoperative, one patient in the stentless valve group, died of a major hemorrhage, on the intensive care ward. He required tracheotomy due to prolonged ventilation and developed fatal intratracheal bleeding. In the mechanical valve group there were no early, but one late death. At 6 months postoperatively the patient had a gastrointestinal bleeding and died before reaching the hospital. Rethoracotomy for bleeding had to be performed in one patient in the stentless valve group, due to cardiac tamponade. All other patients had an uneventful recovery and were discharged from hospital. At follow up, there was one additional gastrointestinal bleeding complication in the mechanical valve group. One patient in this group developed a mild paravalvular leak. He remains under close observation by his cardiologist and so far no significant hemolysis or increase in regurgitation has occurred. At 8 months postoperatively, one patient in the stentless valve group suffered a stroke. There were no other valve related complications in this group.

Echocardiographic evaluation showed no significant difference in hemodynamic performance or rate and extent of LV-mass regression between the groups. All relevant data are summarized in table 3.

### Group III (*stentless vs. stented bioprosthesis, age > 75 years*)

Demographic data and clinical characteristics were comparable between the groups (table 1). The cross-clamp and total cardiopulmonary bypass times were significantly longer in the stentless valve group. There were two not-valve related early deaths (pneumonia, septicemia) and one late death (ruptured abdominal aortic aneurysm) in the stented valve group. The other intra- and postoperative outcomes were comparable between the groups. One patient in each group suffered a stroke. There was one anticoagulation-related bleeding complication during the early postoperative phase in the stented valve group. One patient in each group developed endocarditis and were reoperated.

Echocardiographic evaluation at discharge, 6 and 12 months postoperatively, again did not reveal any significant differences in the rate and completeness of LV mass regression. The hemodynamic performance of the two bioprosthesis was comparable, with regards to mean transvalvular gradient, effective orifice area and ejection fraction. All relevant data are shown in table 3.

A summary of clinical status at the follow up examination is given in table 4.

**Table 4:** Clinical status at 12 months postoperatively

	ROSS n = 20	MIRA n = 20	PRIMA PLUS n = 20	MIRA n = 20	PRIMA PLUS n = 20	PERI- MOUNT n = 20
NYHA I - II	n = 20	n = 20	n = 18	n = 17	n = 18	n = 16

NYHA III - IV	n = 0	n = 0	n = 1	n = 2	n = 2	n = 1

Mean systolic RR (mmHg)	129 ± 21	123 ± 19	128 ± 15	135 ± 16	132 ± 15	136 ± 18

Sinus rhythm	n = 20	n = 18	n = 18	n = 19	n = 18	n = 15

Atrial Fibrillation	n = 0	n = 2	n = 1	n = 0	n = 2	n = 2

Mortality	n = 2	n = 1	n = 1	n = 1	n = 0	n = 3

SAE	n = 2	n = 1	n = 1	n = 2	n = 2	n = 3

## Discussion

Although AVR can be performed with low perioperative and postoperative risk, the optimal substitute for the native aortic valve has not been found. A significant postoperative regression of hypertrophy and improvement in LV-function is achieved by most prostheses. However residual LV-hypertrophy is common after AVR and impairs LV diastolic function which can lead to late congestive cardiac failure. He and colleagues reported on a cohort of patients where incomplete regression of LV hypertrophy significantly reduced 10 year survival [3]. Unresolved LV hypertrophy not only increases mortality but also compromises quality of life and increases morbidity [4]. Michel and colleagues, showed an increased incidence and severity of ventricular arrhythmias in patients with LV hypertrophy after aortic valve replacement [5]. Persistent hypertrophy may be due to the obstructive nature of the valve itself, host related factors or due to patient prosthesis mismatch. Valve-related left ventricular pressure increase is an important reason for incomplete regression of cellular hypertrophy and the development of increased interstitial fibrosis postoperatively [6].

Therefore, one could argue that to achieve an optimal postoperative result, prosthesis has to be chosen that incorporates least obstructiveness with best hemodynamic performance. We would expect a subsequent faster and more complete regression of LV-hypertrophy with the use of such prostheses. Based on valve performance and its effects on regression of LV-hypertrophy, the current study was designed to provide some rationale to select the optimal valve substitute for patients in a certain age group.

The beneficial effects of a less obstructive valve (pulmonary autografts, stentless valves) have often been demonstrated [7-10]. However, in the case of pulmonary autografts there are none, and for stentless valves there are only four randomized trials, comparing their performance to more obstructive valves (stented bioprosthesis, mechanical valves).

In our study, the pulmonary autografts had significantly lower transvalvular gradients than the mechanical valves. From our understanding of the pathophysiology of aortic valve stenosis, we would have expected a significant difference in the regression of left ventricular hypertrophy between the two valve substitutes. However, in this randomized group of patients, left ventricular mass regression was similar in both groups at 6 and 12 months, despite the superior hemodynamic performance of the pulmonary autografts. Significant regression of left ventricular hypertrophy has been reported in literature after aortic valve replacement with both substitutes [10-13]. The 12 month postoperative follow-up period, also seems to be sufficient to assess the regression of left ventricular hypertrophy. Several authors have demonstrated that no difference in left ventricular mass regression is found between 1 year and 3 years of follow up [9,13,14]. At this point one can ask if the statistical difference in transvalvular gradients was clinically relevant. Considering that a peak systolic gradient of up to 20 mmHg can be considered physiologic, we noted that in both groups the peak gradients lay below the 20 mmHg mark (pulmonary autografts 4.8 mmHg and mechanical valves 16.2 mmHg). Interestingly, Walter and colleagues reported a significant difference in the rate of left ventricular mass regression in patients with peak transvalvular pressure gradients of 16.7 mmHg after stentless versus 20.1 mmHg after stented aortic valve replacement, in a randomized cohort of 180 patients [15]. In group II (mechanical vs. stentless) and in group III (stentless vs. stented aortic valve replacement) there was no significant difference in transvalvular gradients and therefore, no significant difference in the rate and completeness of left ventricular mass regression. All valves implanted showed good hemodynamic performance with peak gradients below 20 mmHg. A number of non-randomized studies have been published, especially comparing stentless with stented bioprosthesis. Jin and co-workers evaluated the regression of left ventricular mass in a large numbers of patients after aortic valve replacement with different types of valve substitutes. They found that patients with stentless valves or homografts had a greater reduction of left ventricular mass than patients who received a stented bioprosthesis or mechanical valve. They also found that left ventricular mass regression had been completed at 6 months postoperatively in patients with stentless valves, whereas regression had not been completed after 12 months in patients with stented or mechanical valves [9]. De Paulis and colleagues compared stented, stentless and mechanical valves and although stentless valves resulted in a significantly lower peak systolic gradient, there was no significant difference in the rate and completeness of left ventricular mass regression after 12 months [10].

Cohen et al. also conducted a prospective randomized trial. Ninety-nine patients were randomly assigned to stentless or stented valves. Interestingly, they reported no difference in the rate and completeness of left ventricular mass regression and also no statistically significant difference in hemodynamic performance between these valves [16].

We would expect an aortic valve substitute with optimized hemodynamic performance and minimal or no residual postoperative gradient as in pulmonary autografts or stentless valves to result in better left ventricular remodelling and function. At 12 months follow-up however, looking at left ventricular mass regression we could not distinguish between patients receiving less or more obstructive valve substitutes.

In conclusion we would like to state that significant regression of left ventricular hypertrophy can be achieved by all tested valve substitutes. Based on the findings of our prospective randomized trial, we can recommend the use of any tested valves in the respective age groups. The personal preference and skill of the implanting surgeon will continue to play an important role in choosing a certain valve type.

## Conclusion

However, the overall complexity of pulmonary autograft and stentless valve implantation, with its prolonged cross clamping times might under these circumstances not be justifiable if, as we found, the same results can be achieved with standard stented and mechanical valves.

## List of abbreviations

BSA: Body Surface Area; CABG: Coronary Artery Bypass Grafting; EF: Ejection Fraction; IVS: Interventricular Septal Thickness; LV: Left Ventricular; LVW: Left Ventricular Posterior Wall Thickness; NYHA: New York Heart Association.

## Competing interests

The authors declare that they have no competing interests.

## Authors' contributions

MD has made substantial contributions to conception, design, acquisition, analysis and interpretation. JPW has made substantial contributions to data acquisition. AHK has been involved in drafting the manuscript and revising it critically for important content; AM has given final approval of the study design. All authors read and approved the final manuscript.
